# A Fragmentation behavior-guided UHPLC-Q-Orbitrap HRMS method for the quantitative analysis of 26 perfluoroalkyl substances and their alternatives in water

**DOI:** 10.1371/journal.pone.0335264

**Published:** 2025-11-03

**Authors:** Guojian Shao, Tao Liu, You Xia, Peng Zhang, Ye Wang

**Affiliations:** Huzhou Center for Disease Control and Prevention, Key Laboratory of Emergency Detection for Public Health, Huzhou, China; Cairo University, EGYPT

## Abstract

Perfluoroalkyl substances (PFASs), including newly introduced alternatives, are of global concern due to their environmental persistence, bioaccumulation, and potential health risks. We developed and validated a sensitive, selective UHPLC–Q-Orbitrap HRMS method for the comprehensive analysis of 26 PFASs—including perfluorocarboxylic acids (PFCAs), perfluorosulfonic acids (PFSAs), and emerging substitutes—in diverse water matrices. Sample preparation used WAX solid-phase extraction with isotope-labeled internal standards and separation on a BEH C18 column; quantitative performance was assessed under Full MS, targeted SIM, and PRM. The method achieved excellent linearity, sub-ng/L detection capability, and robust recoveries and precision across matrices, with PRM offering the best balance of sensitivity and selectivity. Characteristic fragmentation patterns, like decarboxylation and desulfonation, supported structural confirmation and resulted in diagnostic fragments that facilitated isomer differentiation and increased the reliability of identifying low-abundance compounds. This fragmentation-guided UHPLC-HRMS approach enables high-confidence detection and quantification of both legacy and novel PFASs and provides a practical tool for environmental monitoring and risk assessment.

## 1. Introduction

Perfluoroalkyl and polyfluoroalkyl substances (PFASs) constitute a broad class of synthetic organic compounds defined by fully or partially fluorinated carbon chains. The strong carbon–fluorine bond confers exceptional chemical and thermal stability, surfactant properties, and dual hydrophobic and lipophobic characteristics [1,2]. These attributes have facilitated their widespread use in industrial processes and consumer products, including textiles, paper, packaging, coatings, construction materials, and medical devices [3,4]. However, the same chemical stability that makes PFASs industrially valuable also underlies their environmental persistence, bioaccumulation potential, and resistance to degradation [5]. Consequently, PFASs have been widely detected across environmental matrices, particularly in drinking water, raising serious concerns for both environmental and public health [6,1]. In response to increasing regulatory restrictions and public awareness surrounding long-chain legacy PFASs, emerging short-chain alternatives—such as hexafluoropropylene oxide dimer acid (HFPO-DA), 6:2 fluorotelomer sulfonic acid (6:2 FTSA), chlorinated polyfluoroether sulfonic acid (Cl-PFESA), perfluorobutanesulfonic acid (FBSA), and dodecafluoro-3H-4,8-dioxanonanoic acid (DONA)—have been introduced as replacements [7–9]. However, their environmental occurrence, fate, and toxicity remain poorly understood, necessitating sensitive and robust analytical methods for simultaneous detection and quantification.

From regulatory and toxicological perspectives, substantial evidence confirms that many legacy per- and polyfluoroalkyl substances (PFASs), such as perfluorooctanoic acid (PFOA) and perfluorooctane sulfonate (PFOS), possess persistence, bioaccumulation potential, and toxicity (PBT) [10,11]. These compounds are associated with serious health risks, including endocrine disruption, immunotoxicity, and developmental toxicity [12]. As a result, strict regulatory frameworks have been implemented across multiple countries and regions. For example, under the European Union’s REACH regulation, PFOS is classified as a restricted substance [13]. In the United States, the Environmental Protection Agency (EPA) has introduced the “PFAS Strategic Roadmap” to establish emission limits for multiple PFASs [14]. In response to these regulations, traditional PFASs have been gradually phased out and replaced with structurally similar but toxicologically understudied alternatives, such as HFPO-DA and DONA. However, global monitoring data indicates that although concentrations of legacy PFASs are declining in some regions, the total PFAS burden in aquatic environments remains largely unchanged [1]. Instead, an increasing variety of novel PFASs has been detected, leading to greater chemical complexity in environmental matrices [15]. In China, 6:2 Cl-PFESA was detected in Haihe River samples from Tianjin at a median concentration of 6.56 ng/L [16]. In the coastal regions of Bohai Bay, HFPO-DA concentrations ranged from non-detectable to 3.99 ng/L [17,18]. Notably, HFPO-DA concentrations in surface waters of the North Sea were approximately three times higher than those of PFOA [19]. Similarly, in the North American Great Lakes, perfluoroethylcyclohexane sulfonate (PFECHS) was detected at concentrations ranging from 0.16 to 5.65 ng/L, comparable to those of PFOS (0.16–5.51 ng/L) [20]. These findings reflect a paradigm shift in PFAS contamination, transitioning from legacy compounds to emerging alternatives and more complex mixtures.

However, current analytical techniques face significant limitations in addressing the growing complexity of PFAS contamination. The mainstream detection of PFASs continues to rely predominantly on liquid chromatography–tandem mass spectrometry (LC-MS/MS) [21,22] and gas chromatography–mass spectrometry (GC-MS) [23,24]. While these techniques provide high sensitivity for conventional PFAS quantification, they show clear limitations in distinguishing isomers and identifying unknown alternatives. PFASs exhibit a wide variety of structures and positional isomers [25]. Conventional methods depend on retention time and individual precursor or fragment ions, which often fail to differentiate positional isomers such as PFOS and Cl-PFESA. In addition, many emerging alternatives possess novel structures, and the lack of analytical standards or known fragmentation patterns can result in both false negative and false positives. Furthermore, due to their colorless nature, high polarity, and thermal stability, PFASs are poorly suited for in-situ analysis using GC-MS [26]. This further hampers the comprehensive characterization of their environmental distribution and behavior. Recently, paper spray-based mass spectrometry (PS-MS) has emerged as a rapid and minimally preparative alternative for PFAS screening in water, wastewater, and food packaging materials, showing promise for on-site environmental applications [27].

As PFAS contamination becomes increasingly complex, fragmentation-based quantification strategies are gaining importance [28]. Unlike conventional methods that depend solely on precursor ion intensities, this approach examines PFAS-specific fragmentation patterns generated via collision-induced dissociation (CID). This enables improved identification of isomers and unknown substitutes through fragment library development and specificity profiling. For instance, Cl-PFESA isomers with similar retention times can be distinguished by variations in fragment abundances (e.g., CF_2_, SO_3_^−^) and fragmentation pathways. The integration of high-resolution parallel reaction monitoring (PRM) and data-independent acquisition (DIA) techniques further enhance structural specificity and quantification accuracy [29,30]. In this study, we established a UHPLC-Q-Orbitrap HRMS method for the simultaneous detection of 26 PFASs and their emerging alternatives in water, achieving detection limits as low as 0.02 ng/L, and validated its performance in real-world samples. This technique provides a robust platform for high-throughput, high-resolution PFAS analysis, enabling comprehensive environmental profiling and risk assessment.

## 2. Materials and methods

### 2.1. Chemicals and reagents

A quantitative analytical method was established to simultaneously detect 26 target PFAS compounds and 14 internal standards. Specifically, a methanol-based mixture containing 23 PFAS standards (5 μg/mL; Alta Scientific Co., Ltd., Tianjin, China), perfluoro(2-methyl-3-oxahexanoic acid) (HFPO-DA, ≥ 94.9%; Dr. Ehrenstorfer GmbH, Augsburg, Germany), perfluorobutanesulfonamide (FBSA, ≥ 98.3%; Dr. Ehrenstorfer GmbH, Augsburg, Germany), and 6:2 fluorotelomer sulfonic acid (6:2 FTSA, ≥ 95%; Cambridge Isotope Laboratories, Inc., Tewksbury, MA, USA), along with a methanol solution of 14 isotope-labeled internal standards (5 μg/mL; Alta Scientific Co., Ltd., Tianjin, China), was used (detailed abbreviations, full names, and types are shown in S1 Table). Additional reagents included HPLC-grade acetonitrile and methanol (Merck KGaA, Darmstadt, Germany), formic acid and ammonium hydroxide solution (Shanghai Macklin Biochemical Co., Ltd., Shanghai, China), ammonium formate (CNW Technologies GmbH, Düsseldorf, Germany), ammonium acetate (Shanghai Macklin Biochemical Co., Ltd., Shanghai, China), and ultrapure water produced by a Milli-Q system (Merck Millipore, Burlington, MA, USA). Solid-phase extraction was carried out using Oasis WAX cartridges (6 cc, 150 mg; Waters Corporation, Milford, MA, USA). PTFE syringe filters (13 mm, 0.22 μm) were obtained from ANPEL Laboratory Technologies (Shanghai) Inc., Shanghai, China.

### 2.2. Standard solution preparation

Weighed 1.0 mg each of HFPO-DA, FBSA, and 6:2FTSA standards were dissolved in methanol and diluted to 10 mL in volumetric flasks. The solutions were thoroughly mixed to prepare 100 mg/L stock standard solutions. Subsequently, 500 μL each of HFPO-DA, FBSA, and 6:2FTS standard solutions (10 mg/L), together with 1.00 mL of a 23-compound perfluorinated standard solution (5 μg/mL), were transferred into 10 mL volumetric flasks containing methanol and diluted to volume. This procedure yielded a mixed working standard solution containing 26 perfluorinated compounds at 500 μg/L. Similarly, 1.00 mL of a 14-compound perfluorinated internal standard solution (5 μg/mL) was diluted to 10 mL with methanol to prepare a mixed internal standard working solution at 500 μ/L. Aliquots of 4.0, 20.0, 100.0, 200.0, and 400.0 μL of the mixed working standard solution (500 μg/L) were individually transferred into 10 mL volumetric flasks containing a 1:1 methanol-water mixture. Then, 100 μ of the mixed internal standard working solution (500 μ/L) was added. The solutions were then diluted to volume and thoroughly mixed. This produced a series of standard solutions containing 26 perfluorinated compounds at concentrations of 0.2, 1.0, 5.0, 10.0, and 20.0 μ/L, with internal standards maintained at 5.0 μg/L.

### 2.3. Sample collection

Sampling was conducted at 18 designated sites across nine municipal drinking water treatment plants in Huzhou, covering water sources from lakes, reservoirs, rivers, and streams. Both raw water (untreated source water collected at the plant intake) and treated water (finished tap water after standard treatment processes) were collected, detailed information is available in S2 Table. The sampling procedure strictly adhered to the quality assurance guidelines of USEPA Method 537.1. High-density polyethylene (HDPE) bottles were used as sampling containers. They were sequentially rinsed with ultrapure water and a 50:50 (v/v) methanol/water solution, then air-dried naturally. Field personnel wore powder-free nitrile gloves throughout sampling and adhered to a standardized pre-sampling protocol, which included flushing the water source for three minutes before sample collection. Samples were immediately stored in light-shielded ice boxes maintained at 4 ± 0.5 °C and transported to the laboratory, where pretreatment was completed within 24 hours. Prior to instrumental analysis, samples were stored at –80 °C to preserve stability. In total, 18 water samples (raw and treated water from nine treatment plants) were collected and analyzed using UHPLC-Q-Orbitrap HRMS, all of which met strict quality assurance requirements and were considered valid for analysis.

All samples were collected between 2023 and 2024, with the entire sampling process authorized by the Huzhou Center for Disease Control and Prevention.

### 2.4. Sample extraction

Enrichment and purification were performed using Oasis WAX solid-phase extraction (SPE) cartridges with an automated SPE system. The cartridges were sequentially conditioned with 5 mL of 0.1% ammonium hydroxide in methanol, 7 mL methanol, and 10 mL ultrapure water. Ten microliters of mixed internal standard working solution (100 μg/L) were added to 200 mL of water sample and mixed thoroughly. The sample was loaded onto the WAX cartridge at a flow rate of 8 mL/min. The cartridge was then washed sequentially with 5 mL of 25 mmol/L ammonium acetate-acetic acid buffer (pH 4) and 12 mL of ultrapure water, followed by drying under nitrogen for 15 minutes. The analytes were eluted sequentially with 5 mL methanol and 7 mL of 0.1% ammonium hydroxide in methanol into 15 mL polypropylene centrifuge tubes. The eluate was concentrated to near dryness under nitrogen at 40 °C, reconstituted in 200 μL of 1:1 methanol-water solution, centrifuged at 10,000 rpm, and stored at 4 °C until analysis.

### 2.5. Instrumental analysis

This study utilized a Vanquish UHPLC system (Thermo Scientific, USA) coupled with a Q Exactive Orbitrap high-resolution mass spectrometer (Thermo Scientific, USA). Chromatographic separation was carried out on an Acquity UPLC BEH C18 column (1.7 μm, 2.1 × 100 mm). The mobile phase comprised 2 mmol/L ammonium formate in water (A) and methanol (B). The gradient elution program was: 0–1 min, 20% B; 1–8 min, linear increase to 90% B; 8–12 min, 90% B; 12–12.5 min, decrease to 20% B; 12.5–15 min, 20% B. The flow rate was 0.3 mL/min, the column temperature 40 °C, and the injection volume 10 μL. Mass spectrometric analysis employed a heated electrospray ionization (HESI) source in negative ion mode. The ion transfer tube temperature was 320 °C, and the spray voltage 3.0 kV. Sheath and auxiliary gas flow rates were 45 and 105 arbitrary units, respectively, with an auxiliary gas temperature of 350 °C. Parallel reaction monitoring (PRM) mode was used with a scan range of m/z 100–1000. Mass resolution was 35,000, automatic gain control (AGC) target 1 × 10^5^, and maximum injection time 100 ms. Data acquisition and quantitative analysis were performed using Xcalibur software (version 4.4, Thermo Fisher Scientific, USA).

### 2.6. Method validation

The analytical method was validated in accordance with internationally recognized guidelines for environmental contaminant analysis, including the United States Environmental Protection Agency (USEPA) Method 537.1 and the European Commission guidance documents SANTE/12682/2019. Validation procedures were designed to ensure the method’s reliability for trace-level detection of per- and polyfluoroalkyl substances (PFASs) in complex water matrices. Key validation parameters included linearity, limits of detection (LOD), limits of quantification (LOQ), accuracy, and precision. Linearity was assessed by constructing calibration curves using matrix-matched standards across a range of concentrations, with correlation coefficients evaluated to ensure acceptable linear response. LOD and LOQ were estimated based on signal-to-noise criteria and further confirmed through spiked blank samples.

The Analytical Method Committee recommends evaluating the linearity of a calibration function using the F-test [31], in which an appropriate number of concentration levels (I) are selected, each measured in J replicates, and the residual (SS_r_), pure error (SS_ε_), and lack-of-fit (SS_lof_) sums of squares are calculated. The experimentally obtained F_(I−2)/(IJ−I)_ is then compared with the tabulated F value at the 95% confidence level (I–2 and IJ–J degrees of freedom for the numerator and denominator, respectively); fulfillment of F_tabulated_ >F_(I−2)/(IJ−I)_ confirms linearity.

Accuracy was evaluated through recovery experiments using replicate samples spiked at multiple concentration levels, while intra- and inter-day precision were assessed by calculating the relative standard deviations (RSDs) of repeated measurements. Quality control samples, procedural blanks, and solvent blanks were included in each analytical batch to monitor potential contamination and ensure data reliability. All validation results met the required acceptance criteria, demonstrating the method’s suitability for quantitative analysis of target analytes in water matrices.

## 3. Results and discussion

### 3.1. Optimization of chromatography conditions

**Fig 1:**
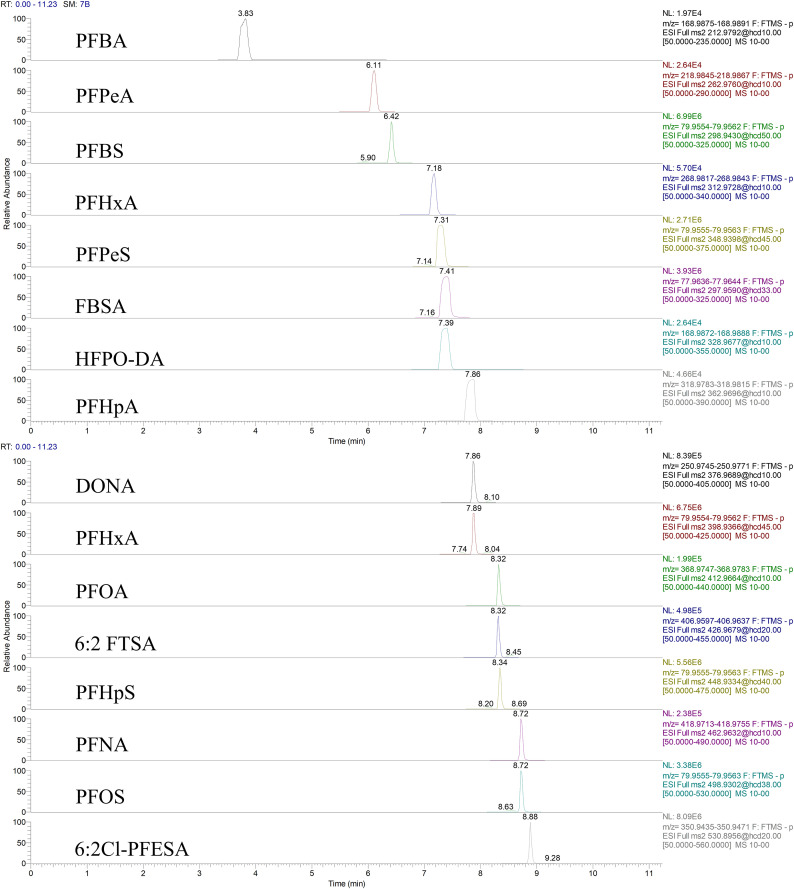
Representative chromatographic separation of 16 target PFAS compounds under optimized conditions. Chromatographic peaks and retention times are shown for each PFAS monitored in their specific ion channels. Information on the other 10 PFAS is provided in S1 Fig.

To optimize chromatographic conditions for the separation of 26 per- and polyfluoroalkyl substances (PFASs) and their emerging alternatives, three reversed-phase columns were evaluated: Acquity UPLC BEH C18, Acquity UPLC HSS T3, and Thermo Scientific Accucore aQ C18. Several mobile phase compositions were tested, including water–methanol, 1 mmol/L ammonium formate in methanol–water, 2 mmol/L ammonium formate in methanol–water, and 5 mmol/L ammonium formate in methanol–water systems. In addition, three reconstitution solvents—methanol, acetonitrile, and methanol/water (1:1, v/v)—were compared to assess their effects on peak shape and solvent compatibility. Preliminary analysis using a mixed standard solution (10 μg/L) of target PFASs and alternatives indicated that the Acquity UPLC BEH C18 column provided superior separation efficiency across the tested compounds. The mobile phase containing 2 mmol/L ammonium formate in methanol–water exhibited the highest overall sensitivity. Among the reconstitution solvents, methanol/water (1:1, v/v) yielded symmetric peak shapes with minimal solvent-related artifacts. The representative chromatographic separation of the 26 target compounds under the optimized conditions is illustrated in Fig 1.

### 3.2. Optimization of mass spectrometry conditions

Perfluorocarboxylic acids (PFCAs), perfluorosulfonic acids (PFSAs), and their substitutes were analyzed by tandem mass spectrometry (MS/MS) targeting [M–H]^−^ ions, using a quadrupole-electrostatic field Orbitrap high-resolution mass spectrometer. In PFCAs, the carboxyl group in the parent ion readily undergoes decarboxylation, yielding the characteristic fragment ion [M–H–CO_2_] ^−^. Subsequent cleavage of varying numbers of C–C bonds produce fragment ions of the type [C_n_F_2n+1_]^−^. For example, perfluorodecanoic acid (PFDA) produces a parent ion [M–H]^−^ at m/z 512.9600, which undergoes decarboxylation to form the characteristic fragment ion [M–H–CO_2_]^−^ at m/z 468.9701. Subsequent C–C bond cleavages yield fragment ions including [C^9^F^19^]^−^, [C^6^F_13_]^−^, [C_5_F_11_]^−^, [C_4_F_9_]^−^, and [C_3_F_7_]^−^.

In contrast, PFSAs preferentially lose C_n_F_2n+1_· radicals from the parent ion, generating abundant [·SO_3_]^−^ and rearranged [FSO_3_]^−^ characteristic fragments. Additionally, cleavage of various C–C bonds produce [C_n_F_2n+1_]^−^ fragment ions. For example, perfluorodecanesulfonic acid (PFDS) forms a parent ion [M–H]^−^ at m/z 598.9238. Loss of the C_10_F_21_· radical generates abundant [·SO_3_]^−^ (m/z 79.9558) and rearranged [FSO_3_]^−^ (m/z 98.9543) fragments, along with [C_3_F_7_]^−^ and [C_2_F_5_]^−^ ions resulting from C–C bond cleavage.

**Fig 2:**
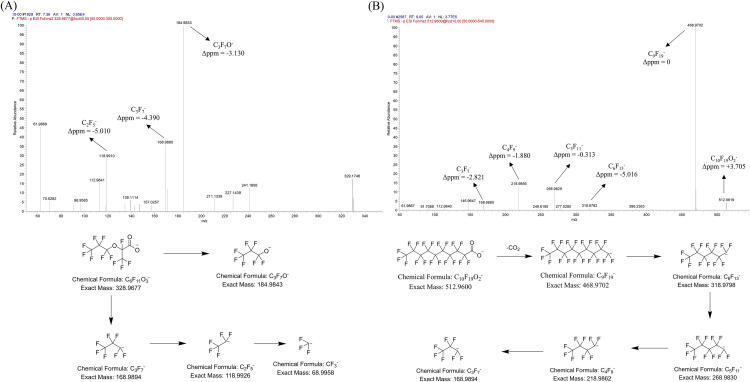
Typical MS/MS spectra and proposed fragmentation pathways of selected PFAS. (A) MS/MS fragmentation pattern and proposed cleavage pathway of HFPO-DA; (B) MS/MS fragmentation pattern and proposed cleavage pathway of PFDA. The figure includes measured and theoretical mass error in parts per million (Δppm) for the fragment ions.

Two PFOA substitutes (HFPO-DA and DONA) and four PFOS substitutes (FBSA, 6:2 FTSA, 6:2 Cl-PFESA, and 8:2 Cl-PFESA) contain ether functional groups that readily undergo either bond cleavage. This produces characteristic [R–O]^−^ and [R]^−^ fragment ions. For instance, HFPO-DA produces a parent ion [M–H]^−^ at m/z 328.9677. Ether bond cleavage yields characteristic fragment ions [R–O]^−^ (m/z 184.9834) and [R]^−^ (m/z 168.9883), followed by C–C bond cleavages generating [C_2_F_5_]^−^ and [CF_3_]^−^ fragments. Tandem MS data are summarized in Table 1, and proposed fragmentation pathways are illustrated in Fig 2. Supplementary Materials S2 Fig shows the MS/MS fragmentation pattern and proposed cleavage pathway of PFDS.

**Table 1:** Optimized mass spectrometric conditions for the quantification of 26 pfas and their substitutes.

Compounds	CAS	Formula	RT (min)	Precursor ion (m/z)	Production (m/z)	CE (eV)	IS
PFBA	375-22-4	C4HF7O2	3.83	212.9792	168.9883	10	^13^C_4_-PFBA
PFPeA	2706-90-3	C5HF9O2	6.11	262.9760	218.9857	10	^13^C_5_-PFPeA
PFBS	375-73-5	C4HF9O3S	6.42	298.9430	79.9558	50	^13^C_3_-PFBS
PFHxA	307-24-4	C6HF11O2	7.18	312.9728	268.983	10	^13^C_5_-PFHxA
PFPeS	2706-91-4	C5HF11O3S	7.31	348.9398	79.9559	45	^13^C_5_-PFHxA
HFPO-DA	13252-13-6	C6HF11O3	7.39	328.9677	168.9883	10	^13^C_5_-PFHxA
FBSA	30334-69-1	4H2F9NO2S	7.41	297.9590	77.9640	33	^13^C_4_-PFHpA
PFHpA	375-85-9	C7HF13O2	7.86	362.9706	318.9798	10	^13^C_4_-PFHpA
DONA	919005-14-4	C7H2F12O4	7.86	376.9689	250.9757	10	^13^C_3_- PFHxS
PFHxS	355-46-4	C6HF13O3S	7.89	398.9365	79.9558	45	^13^C_3_- PFHxS
PFOA	335-67-1	C8HF15O2	8.32	412.9664	368.9765	10	^13^C_8_-PFOA
PFHpS	375-92-8	C7HF15O3S	8.34	448.9333	79.9559	40	^13^C_8_-PFOA
6:2FTSA	27619-94-9	C8H4F13NaO3S	8.32	426.9674	406.9615	20	^13^C_8_-PFOA
PFNA	375-95-1	C9HF17O2	8.72	462.9632	418.9743	10	^13^C_9_-PFNA
PFOS	1763-23-1	C8HF17O3S	8.72	498.9308	79.9558	38	^13^C_8_-PFOS
6:2Cl-PFESA	73606-19-6	C8ClF16KO4S	8.88	530.8956	350.9452	20	^13^C_8_-PFOS
PFNS	68259-12-1	C9HF19O3S	9.06	548.9270	79.9558	35	^13^C_6_-PFDA
PFDA	335-76-2	C10HF19O2	9.05	512.9600	468.9701	10	^13^C_6_-PFDA
PFUdA	2058-94-8	C11HF21O2	9.34	562.9568	518.9674	10	^13^C_7_-PFUdA
PFDS	335-77-3	C10HF21O3S	9.32	598.9238	79.9558	35	^13^C_7_-PFUdA
8:2Cl-PFESA	83329-89-9	C10ClF20KO4S	9.41	630.8895	450.9390	15	^13^C_7_-PFUdA
PFDoA	307-55-1	C12HF23O2	9.56	612.9537	568.9641	10	^13^C_2_-PFDoA
PFTrDA	72629-94-8	C13HF25O2	9.77	662.9504	618.9609	15	^13^C_2_-PFTrDA
PFTeDA	376-06-7	C14HF27O2	9.96	712.9473	668.9577	10	^13^C_2_-PFTeDA
PFHxDA	67905-19-5	C16HF31O2	10.37	812.9409	768.9514	10	^13^C_2_-PFTeDA
PFOdA	16517-11-6	C18HF35O2	10.92	912.9345	868.9446	10	^13^C_2_-PFTeDA

This study compared three data acquisition modes—Full MS, Targeted-SIM, and PRM—using the Orbitrap high-resolution mass spectrometer. Targeted-SIM exhibited fewer data points per peak, poor peak shapes, and low quantitative reproducibility. Full MS mode, which analyzes all ions simultaneously, exhibited elevated baseline noise and reduced sensitivity. Conversely, PRM mode employed segmented scanning with ±0.5 min acquisition windows, yielding over eight data points per peak with symmetrical peak shapes, high sensitivity, and excellent reproducibility (S3 Fig). Therefore, PRM was chosen for the quantitative analysis of 26 perfluorinated compounds. Detailed fragment ions and acquisition parameters are listed in Table 1.

### 3.3. Method Validation

#### 3.3.1. LOD, LOQ and linear range.

Quantification was conducted using UPLC coupled with quadrupole-Orbitrap high-resolution mass spectrometry (UHPLC-Q-Orbitrap HRMS). Calibration curves were generated by plotting analyte concentrations (x-axis) against the product of the analyte-to-internal standard peak area ratio and the internal standard concentration (y-axis). Method validation involved spiking ultrapure water at 1.0 ng/L, followed by six replicate measurements using the sample preparation procedure described in Section 2.2. The method demonstrated excellent linearity for all 26 PFAS (R^2^ > 0.99), with detection limits (LODs) ranging from 0.02 to 0.2 ng/L and quantification limits (LOQs) from 0.06 to 0.6 ng/L. Detailed information on the linear range, LODs, and LOQs is provided in Table 2.

**Table 2:** Linear ranges, limits of detection (LODs), and limits of quantification (LOQs) for 26 PFASs and their alternatives.

Compounds	Linear range(μg/L)	Regression equation	R^2^	LOD(ng/L)	LOQ(ng/L)
PFBA	1.0～20	Y = 0.08637X - 0.03600	0.9994	0.2	0.6
PFPeA	0.2～20	Y = 0.01029X - 0.009238	0.9998	0.2	0.6
PFBS	0.2～20	Y = 0.1116X + 0.01691	0.9998	0.02	0.06
PFHxA	0.2～20	Y = 0.1024X-0.009931	0.9988	0.02	0.06
PFPeS	0.2～10	Y = 8.1727X + 0.3959	0.9997	0.02	0.06
HFPO-DA	0.2～20	Y = 0.01346X-0.004076	0.9997	0.02	0.06
FBSA	0.2～20	Y = 3.5356X-0.01291	0.9995	0.02	0.06
PFHpA	0.2～20	Y = 0.09724X-0.002587	0.9992	0.02	0.06
DONA	0.2～20	Y = 0.6623X-0.01714	0.9989	0.02	0.06
PFHxS	0.2～20	Y = 0.1117X + 0.02483	0.9985	0.02	0.06
PFOA	0.2～20	Y = 0.08905X + 0.001332	0.9984	0.02	0.06
PFHpS	0.2～20	Y = 2.4879X-0.001287	0.9992	0.02	0.06
6:2FTSA	0.2～10	Y = 0.1984X-0.01572	0.9968	0.02	0.06
PFNA	0.2～20	Y = 0.09053X + 0.001235	0.9983	0.02	0.06
PFOS	0.2～20	Y = 0.1155X + 0.003238	0.9985	0.02	0.06
6:2Cl-PFESA	0.2～20	Y = 0.2260X + 0.005032	0.9992	0.02	0.06
PFNS	0.2～10	Y = 0.5627X-0.007492	0.9972	0.02	0.06
PFDA	0.2～20	Y = 0.9151X-0.0005316	0.9982	0.02	0.06
PFUdA	0.2～20	Y = 0.9988X-0.009644	0.9988	0.02	0.06
PFDS	0.2～20	Y = 0.4706X + 0.02052	0.9967	0.02	0.06
8:2Cl-PFESA	0.2～20	Y = 0.03039X + 0.00007281	0.9994	0.02	0.06
PFDoA	0.2～20	Y = 0.08742X + 0.002574	0.9974	0.02	0.06
PFTrDA	0.2～10	Y = 0.03162X + 0.00009824	0.9997	0.02	0.06
PFTeDA	0.2～10	Y = 0.08930X + 0.0003450	0.9957	0.02	0.06
PFHxDA	0.2～20	Y = 0.06434X-0.001564	0.9994	0.02	0.06
PFOdA	0.2～20	Y = 0.03345X + 0.04086	0.9998	0.2	0.6

As shown in S3 Table, using PFOS as an example, the lack-of-fit analysis demonstrated that the calculated Fisher ratio (F = 2.36) was lower than the critical value at the 95% confidence level (F3,10 = 3.71), indicating that the deviation between the experimental data and the fitted regression line is attributable to random error rather than systematic lack of fit. This suggests that the selected linear model adequately describes the calibration relationship across the tested concentration range. Furthermore, the high coefficient of determination (R^2^ = 0.9985) supports the excellent goodness-of-fit, confirming both the precision and linearity of the calibration curve for PFOS under the established analytical conditions.

#### 3.3.2. Accuracy and precision.

Ultrapure water was used as the blank matrix in spike-and-recovery experiments. Samples were spiked at three concentration levels: 1.0, 5.0, and 10.0 ng/L. Six replicate samples were prepared and analyzed for each concentration level, following the protocol described in Section 2.2. At 1.0 ng/L, recoveries ranged from 66.3% to 119.2%, with relative standard deviations (RSDs) of 1.3–7.3%. At 5.0 ng/L, recoveries ranged from 72.5% to 104.1%, with RSDs of 1.1–6.1%. At 10.0 ng/L, recoveries ranged from 62.8% to 96.4%, with RSDs ranging from 1.4% to 6.5%. Detailed recovery and precision data are provided in Table 3.

**Table 3:** Accuracy and precision of 26 PFASs and their alternatives (n = 6).

Compounds	Sample background(ng/L)	Spiked at 1.0 ng/L		RSD(%)	Spiked at 5.0 ng/L		RSD(%)	Spiked at 10 ng/L		RSD (%)
		Average (ng/L)	Average recovery(%)		Average (ng/L)	Average recovery		Average (ng/L)	Average recovery(%)	
PFBA	n.d.	1.04	104.9	5.2	5.00	99.9	3.9	9.40	94.0	3.8
PFPeA	n.d.	0.92	91.6	4.4	4.12	82.5	3.7	8.34	83.3	3.2
PFBS	n.d.	0.98	97.6	1.3	4.72	94.2	1.7	8.94	89.4	1.6
PFHxA	n.d.	1.00	99.1	2.9	4.68	93.4	2.4	8.92	89.1	1.4
PFPeS	n.d.	0.74	74.3	3.1	4.04	80.8	3.6	8.74	87.4	2.8
HFPO-DA	n.d.	1.04	103.8	2.3	4.38	87.6	1.8	8.98	89.7	1.9
FBSA	n.d.	0.68	68.4	5.2	3.62	72.5	4.8	7.46	74.6	4.3
PFHpA	n.d.	1.04	104.3	3.4	4.54	90.6	4.1	9.60	96.0	3.8
DONA	n.d.	0.98	97.2	1.8	4.56	91.2	1.9	9.38	93.8	1.4
PFHxS	n.d.	0.94	93.1	1.9	4.08	81.7	1.1	8.52	85.2	1.4
PFOA	n.d.	0.90	90.8	4.2	4.72	94.2	4.6	9.08	90.7	3.8
PFHpS	n.d.	0.68	67.3	3.7	4.32	86.3	3.7	7.28	72.7	2.4
6:2FTSA	n.d.	0.88	87.9	4.9	4.58	91.7	3.7	9.64	96.4	4.1
PFNA	n.d.	0.94	93.4	3.6	4.72	94.2	3.7	9.14	91.3	2.9
PFOS	n.d.	1.06	105.7	2.6	4.66	93.2	1.3	8.74	87.3	1.5
6:2Cl-PFESA	n.d.	0.74	74.0	3.5	3.96	79.1	2.8	6.28	62.8	2.1
PFNS	n.d.	0.76	76.8	5.7	4.48	89.7	5.4	8.46	84.6	4.6
PFDA	n.d.	0.90	90.4	4.6	4.76	95.3	4.3	9.50	95.0	4.3
PFUdA	n.d.	1.00	99.3	3.2	4.78	95.7	1.5	8.90	89.0	1.7
PFDS	n.d.	0.82	81.3	3.3	3.80	75.9	3.5	7.62	76.1	1.7
8:2Cl-PFESA	n.d.	0.88	87.3	2.4	4.14	82.9	3.2	7.72	77.2	5.3
PFDoA	n.d.	1.20	119.2	5.3	5.02	100.2	5.5	9.38	93.7	4.2
PFTrDA	n.d.	1.04	103.4	6.3	4.82	96.5	4.7	9.12	91.1	4.5
PFTeDA	n.d.	1.18	118.7	4.2	5.20	104.1	3.9	8.90	88.9	3.8
PFHxDA	n.d.	0.66	66.3	6.8	3.64	72.6	6.1	7.48	74.7	6.5
PFOdA	n.d.	0.74	73.5	7.3	4.08	81.6	5.2	8.42	84.1	5.7

n.d.: below the limit of detection.

### 3.4. Comparative evaluation of analytical methods

Growing regulatory oversight and rising public health concerns over PFAS contamination in aquatic environments have made analytical sensitivity and accuracy central to method development. Table 4 summarizes the major platforms currently used for PFAS quantification and their analytical performance. Among these platforms, triple quadrupole mass spectrometry (TQ-MS) remains the most widely used technique, showing consistent recovery and reliable quantitative performance across multiple studies. However, significant variability in detection limits (LODs) and quantification limits (LOQs) remains a major concern. For instance, in a study analyzing 53 PFAS compounds, TQ-MS yielded LODs from 0.28 to 17 ng/L and LOQs up to 21 ng/L [32], indicating inadequate sensitivity for low-level PFAS samples. Similarly, another study targeting 23 PFAS compounds reported a minimum LOD of 0.10 ng/L [33]; however, LOQs still ranged from 0.55 to 1.56 ng/L, limiting the method’s applicability to PFAS substitutes and low background concentrations. Furthermore, a separate study analyzing 24 PFAS compounds reported improved LODs (0.02–1.00 ng/L) but exhibited wide LOQ variability (2.00–50.00 ng/L) [34], highlighting persistent challenges in quantifying PFAS at environmentally relevant trace levels.

**Table 4:** Comparative performance of this study and reported methods for quantitative Analysis of PFAS.

Instrument platform	PFAS compounds	Application matrix	Detection limit (LOD) range	Quantitative limit (LOQ) range	Recovery (%)	Reference
LC–TQMS	53	Water	0.28-17 ng/L	0.4-21 ng/L	66-138	[32]
LC–TQMS	23	Water	0.10-1.21 ng/L	0.6-1.6 ng/L	78–112	[33]
LC–TQMS	24	Water	0.02-1.00 ng/L	2-50 ng/L	88-117	[34]
LC–TQMS	27	Water	0.02–1.45 ng/L	0.1–3.3 ng/L	89-122	[35]
Q Exactive Orbitrap	43	Herbal medicine	10-150 ng/Kg	20-500 ng/Kg	68-121	[36]
Q Exactive Orbitrap	27	Water	0.02-0.22 ng/L	0.06-0.56 ng/L	70-108	[37]
Q Exactive Orbitrap	26	Water	0.02-0.20 ng/L	0.06-0.60 ng/L	73-104	This Study

Compared to conventional mass spectrometry platforms, high-resolution Q Exactive Orbitrap mass spectrometry offers superior detection capabilities for PFAS analysis. One study using this platform reported limits of detection (LODs) ranging from 0.02 to 0.22 ng/L and limits of quantification (LOQs) between 0.06 and 0.56 ng/L for 27 PFASs, with consistent recoveries of 70% to 108% [37]. Another study demonstrated the system’s applicability to complex matrices, reporting LOQs of 20–500 ng/kg for 43 PFASs in herbal samples, highlighting the Orbitrap’s versatility for multi-matrix quantification [36]. In this study, 26 PFASs and their emerging alternatives were analyzed in water samples using the Q Exactive Orbitrap system. The analytical performance was comparable to the highest values reported in the literature. Notably, the method demonstrated clear advantages in detecting trace-level PFAS alternatives, emphasizing its suitability for ultra-trace environmental monitoring.

### 3.5. Quantification of PFASs in water samples

**Fig 3:**
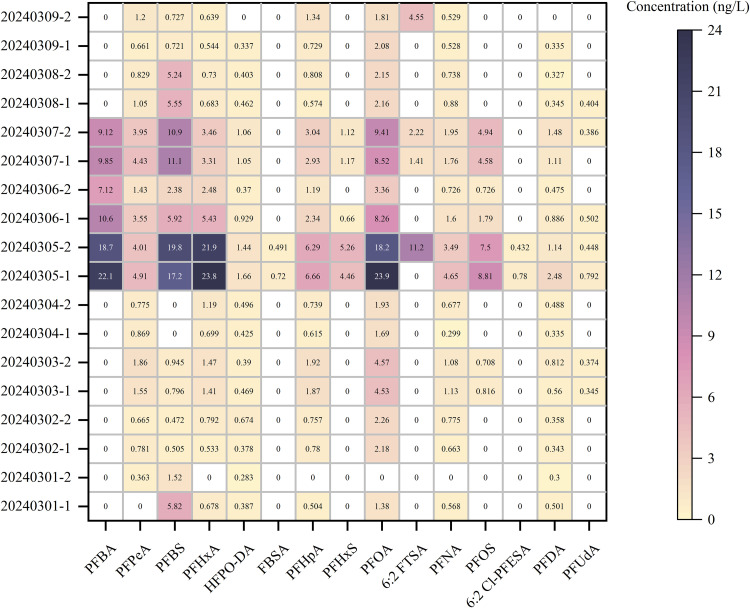
Heatmap of concentrations of 26 PFASs detected in raw and treated water samples collected from nine municipal drinking water treatment plants. The y-axis represents sample IDs, while the x-axis shows the PFAS compounds that were detected. Data labels indicate quantified concentrations (ng/L).

The method was applied to analyze 26 PFASs in both influent (raw) and effluent (treated) water samples collected from nine drinking water treatment plants in Huzhou City. PFASs were detected in all samples, showing significant variability in concentrations and detection frequencies among compounds (Fig 3). PFOA contamination ranged from 1.00 to 23.9 ng/L with a 100% detection frequency; the highest concentration (23.9 ng/L) was found in the Taihu Water Plant. PFOS concentrations ranged from 0.81 to 8.81 ng/L with a 42.1% detection frequency; the highest value (8.81 ng/L) was also detected at the Taihu Water Plant. All measured concentrations were below the limits specified in the Chinese Drinking Water Standard (GB 5749−2022), which sets maximum values of 80 ng/L for PFOA and 40 ng/L for PFOS [38]. Perfluorocarboxylic acids with carbon chain lengths ≥12 were not detected. Short-chain perfluorocarboxylic acids (C4–C7) exhibited relatively high detection rates; PFBA concentrations ranged from 7.1 to 22.1 ng/L with a 33.3% detection frequency. Other short-chain PFASs were detected in all samples, with PFPeA at 0.36–4.91 ng/L, PFHxA at 0.53–23.9 ng/L, PFHpA at 0.36–4.91 ng/L, and PFHxS at 0.57–6.66 ng/L. Among perfluorosulfonates, PFBS ranged from 0.47 to 19.5 ng/L with an 88.9% detection frequency; other short-chain sulfonates were not detected. For other PFAS alternatives, HFPO-DA concentrations ranged from 0.28 to 1.66 ng/L with a 99.4% detection frequency; 6:2 FTSA ranged from 1.41 to 11.2 ng/L (22.2% detection); FBSA ranged from 0.49 to 0.72 ng/L (11.1% detection); and 6:2 Cl-PFESA ranged from 0.43 to 0.78 ng/L (11.1% detection). Neither 8:2 Cl-PFESA nor DONA was detected in any samples.

The widespread detection of PFASs in both source and treated waters across multiple drinking water treatment facilities underscores the pervasive nature of these contaminants in the regional aquatic environment. While all quantified concentrations remained below the regulatory thresholds defined in China’s Drinking Water Standard (GB 5749−2022), the frequent occurrence of legacy PFASs such as PFOA and PFOS—as well as several short-chain and alternative compounds including PFBA, PFBS, HFPO-DA, and 6:2 FTSA—highlights the evolving complexity of PFAS contamination profiles. Consistent with previous research findings, the highest total PFAS burden was observed in the Taihu Lake water supply system [39,40], suggesting potential point sources and limited attenuation through conventional treatment processes.

From a health-risk perspective, EFSA group Tolerable Weekly Intake of 4.4 ng/kg bw/week for the sum of PFOS, PFOA, PFNA, and PFHxS indicates that even low-ng/L drinking-water contributions can be non-negligible when combined with dietary and other sources [41]; in parallel, IARC now classifies PFOA as carcinogenic to humans (Group 1) and PFOS as possibly carcinogenic (Group 2B) [42]. Operationally, the frequent occurrence of short-chain and ether PFAS (e.g., PFBA, PFBS, HFPO-DA, 6:2 FTSA) is notable because these species are more mobile and generally harder to remove by conventional treatment and even by adsorption compared with long-chain analogues, implying earlier breakthrough and higher residuals without optimized barriers [43]. Consistent with this, the highest total PFAS burden observed in the Taihu Lake supply indicates potential upstream point sources and limited attenuation through conventional trains; published trend analyses similarly report persistent PFAS signatures in Taihu despite control actions [39]. Collectively, these findings support continued mixture-aware monitoring, source control, and treatment optimization, even where individual compounds do not exceed domestic reference limits.

## 4. Conclusion

We established a highly sensitive and structurally informative workflow for the simultaneous determination of 26 per- and polyfluoroalkyl substances in environmental waters using UHPLC–Q-Orbitrap HRMS. By integrating fragmentation behavior into method development and employing PRM acquisition, the approach delivers high sensitivity, precision, and structural confidence—particularly for isomeric and low-abundance compounds—while remaining practical across diverse water matrices. By enabling reliable surveillance, early warning, and data-driven decision-making, this workflow supports the delivery of safe drinking water and strengthens the evidence base for responsible management of hazardous chemicals across water systems. Its analytical robustness and transferability provide a practical foundation for utilities and regulators to prioritize interventions, evaluate treatment performance, and guide continuous improvement in water-quality protection.

## Supporting information

S1 Table Abbreviations, full chemical names, classifications, and sources of the 26 per- and polyfluoroalkyl substances (PFASs) analyzed in this study. (DOCX)

S2 Table Detailed characteristics of sampling sites in this study include sample ID, water treatment plant, source water type, location, and disinfection method. (DOCX)

S3 Table Lack-of-fit test results for the calibration curve of PFOS. (DOCX)

S1 Fig Representative chromatographic separation of 10 target PFAS compounds under optimized conditions. Chromatographic peaks and retention times are shown for each PFAS monitored in their specific ion channels.
(DOCX)

S2 Fig MS/MS fragmentation pattern and proposed cleavage pathway of PFDS. (DOCX)

S3 Fig Illustrative example using PFOS in Orbitrap HRMS: Comparison of mass spectra acquired in (a) SIM mode, (b) PRM mode, and (c) Full-MS mode. (DOCX)

S1 File Minimal Dataset. (ZIP)
