# Supplementary material for: A Fragmentation behavior-guided UHPLC-Q-Orbitrap HRMS method for the quantitative analysis of 26 perfluoroalkyl substances and their alternatives in water
Source: PLoS One. 2025 Nov 3;20(11):e0335264. doi: 10.1371/journal.pone.0335264 (PMC12582490; doi:10.1371/journal.pone.0335264)
Supplement: S1 Table — (DOCX) [file pone.0335264.s001.docx]

**Table S1.** Abbreviations, full chemical names, classifications, and sources of the 26 per- and polyfluoroalkyl substances (PFASs) analyzed in this study.

| **Abbreviations** | **Name** | **Supplier** |  |
| --- | --- | --- | --- |
| **Perfluoroalkyl carboxylic acids(PFCAs)** |  |  |  |
| PFBA | Perfluorobutanoic acid | Alta Scientific |  |
| PFPeA | Perfluoropentanoic acid | Alta Scientific |  |
| PFHxA | Perfluorohexanoic acid | Alta Scientific |  |
| PFHpA | Perfluoroheptanoic acid | Alta Scientific |  |
| PFOA | Perfluorooctanoic acid | Alta Scientific |  |
| PFNA | Perfluorononanoic acid | Alta Scientific |  |
| PFDA | Perfluorodecanoic acid | Alta Scientific |  |
| PFUdA | Perfluoroundecanoic acid | Alta Scientific |  |
| PFDoA | Perfluorododecanoic acid | Alta Scientific |  |
| PFTrDA | Perfluorotridecanoic acid | Alta Scientific |  |
| PFTeDA | Perfluorotetradecanoic acid | Alta Scientific |  |
| PFHxDA | Perfluorohexadecanoic acid | Alta Scientific |  |
| PFOdA | Perfluorooctadecanoic acid | Alta Scientific |  |
| **Perfluoroalkyl sulfonic acids (PFSAs)** |  |  |  |
| PFBS | Perfluorobutane sulfonate | Alta Scientific |  |
| PFPeS | Perfluorohexane sulfonate | Alta Scientific |  |
| PFHxS | Perfluorohexane sulfonate | Alta Scientific |  |
| PFHpS | Perfluoroheptane sulfonate | Alta Scientific |  |
| PFOS | Perfluorooctane sulfonate | Alta Scientific |  |
| PFNS | perfluorononanesulfonic acid | Alta Scientific |  |
| PFDS | Perfluorodecane sulfonate | Alta Scientific |  |
| **PFAS precursor** |  |  |  |
| FBSA | Perfluoro-1-butane-sulfonamide | Dr. E |  |
| **Alternative PFASs** |  |  |  |
| DONA | 4,8-Dioxa-3H-perfluorononanoic acid | Alta Scientific |  |
| 6:2 FTSA | 6:2 Fluorotelomer sulfonate | Cambridge |  |
| 6:2Cl-PFESA | 6:2 Chlorinated polyfluoroalkyl ether sulfonic acids | Alta Scientific |  |
| 8:2Cl-PFESA | 8:2 Chlorinated polyfluoroalkyl ether sulfonic acids | Alta Scientific |  |
| HFPO-DA | Perfluoro-2-propoxypropanoic acid | Dr. E |  |
| **Internal Standard (IS)** |  |  |  |
| ^13^C_4_-PFBA | Perfluoro-n-[1,2,3,4-13C4]butanoic acid | Alta Scientific |  |
| ^13^C_5_-PFPeA | Perfluoro-n-[13C5]pentanoic acid | Alta Scientific |  |
| ^13^C_5_-PFHxA | Perfluoro-n-[1,2,3,4,6-13C5]hexanoic acid | Alta Scientific |  |
| ^13^C_4_-PFHpA | Perfluoro-n-[1,2,3,4-13C4]heptanoic acid | Alta Scientific |  |
| ^13^C_8_-PFOA | Perfluoro-n-[13C8]octanoic acid | Alta Scientific |  |
| ^13^C_9_-PFNA | Perfluoro-n-[13C9]nonanoic acid | Alta Scientific |  |
| ^13^C_6_-PFDA | Perfluoro-n-[1,2,3,4,5,6-13C6]decanoic acid | Alta Scientific |  |
| ^13^C_7_-PFUdA | Perfluoro-n-[1,2,3,4,5,6,7-13C7]undecanoic acid | Alta Scientific |  |
| ^13^C_2_-PFDoA | Perfluoro-n-[1,2-13C2]dodecanoic acid | Alta Scientific |  |
| ^13^C_2_-PFTeDA | Perfluoro-n-[1,2-13C2]tetradecanoic acid | Alta Scientific |  |
| ^13^C_3_-PFBS | Sodium perfluoro-1-[2,3,4-13C3]butanesulfonate | Alta Scientific |  |
| ^13^C_3_- PFHxS | Sodium perfluoro-1-[1,2,3-13C3]hexanesulfonate | Alta Scientific |  |
| ^13^C_8_-PFOS | Sodium perfluoro-1-[13C8]octanesulfonate | Alta Scientific |  |
| ^13^C_2_-PFTrDA | Perfluoro-n-[1,2-13C2]tridecanoic acid | Alta Scientific |  |
