# Supplementary material for: A Fragmentation behavior-guided UHPLC-Q-Orbitrap HRMS method for the quantitative analysis of 26 perfluoroalkyl substances and their alternatives in water
Source: PLoS One. 2025 Nov 3;20(11):e0335264. doi: 10.1371/journal.pone.0335264 (PMC12582490; doi:10.1371/journal.pone.0335264)
Supplement: S2 Table — (DOCX) [file pone.0335264.s002.docx]

**Table S2.** Detailed characteristics of sampling sites in this study include sample ID, water treatment plant, source water type, location, and disinfection method.

| **Sample ID** | **Raw water sample** | **Treated water sample** | **Sampling site name** | **Source type** | **District/County** | **Plant type** | **Disinfection method** |
| --- | --- | --- | --- | --- | --- | --- | --- |
| 20240301 | 20240301-1 | 20240301-2 | West Water Plant | Reservoir | Wuxing District | Urban | Chlorine gas |
| 20240302 | 20240302-1 | 20240302-2 | Daixi Water Co., Ltd. | Reservoir | Wuxing District | Rural | Chlorine gas |
| 20240303 | 20240303-1 | 20240303-2 | Changxing Water Co., Ltd. | Reservoir | Changxing County | Urban | Sodium hypochlorite |
| 20240304 | 20240304-1 | 20240304-2 | Anji Shangshe Water Supply Station | Stream | Anji County | Rural | Sodium hypochlorite |
| 20240305 | 20240305-1 | 20240305-2 | Taihu Water Plant | Lake | Nanxun District | Urban | Chlorine gas |
| 20240306 | 20240306-1 | 20240306-2 | Deqing Dakuo Water Purification Co., Ltd. | River (Dongtiaoxi) | Deqing County | Rural | Sodium hypochlorite |
| 20240307 | 20240307-1 | 20240307-2 | Changxing Yongda Water Co., Ltd., Third Plant | River (Xitiaoxi) | Changxing County | Rural | Sodium hypochlorite |
| 20240308 | 20240308-1 | 20240308-2 | Anji Chengxi Water Plant | Reservoir | Anji County | Urban | Sodium hypochlorite |
| 20240309 | 20240309-1 | 20240309-2 | Deqing Huanzhong Water Purification | Reservoir | Deqing County | Urban | Sodium hypochlorite |
