# Supplementary material for: A Fragmentation behavior-guided UHPLC-Q-Orbitrap HRMS method for the quantitative analysis of 26 perfluoroalkyl substances and their alternatives in water
Source: PLoS One. 2025 Nov 3;20(11):e0335264. doi: 10.1371/journal.pone.0335264 (PMC12582490; doi:10.1371/journal.pone.0335264)
Supplement: S3 Table — (DOCX) [file pone.0335264.s003.docx]

**Table S3.** Lack-of-fit test results for the calibration curve of PFOS.

| **Comments** | | | | **X** | **Y** |  |  | $\bar{\mathbf{Y}}$ |  |  |
| --- | --- | --- | --- | --- | --- | --- | --- | --- | --- | --- |
| The amount of analyte (μg/L), the chromatographic analyte/internal standard peak area ratio and its average are designated as X, Y and $\bar{Y}$ respectively. | | | | 0.2 | 0.0225 | 0.0284 | 0.0334 | 0.0281 |  |  |
|  |  |  |  | 1.0 | 0.1054 | 0.1245 | 0.1457 | 0.1252 |  |  |
|  |  |  |  | 5.0 | 0.5836 | 0.6104 | 0.6366 | 0.6102 |  |  |
|  |  |  |  | 10.0 | 1.0172 | 1.1646 | 1.2961 | 1.1593 |  |  |
|  | | | | 20.0 | 2.0425 | 2.1745 | 2.3062 | 2.1744 |  |  |
| The calibration curve was obtained by plotting y vs x.  Proposed linear model by using the reported data.  Reported squared correlation coefficient (R^2^). | | | | ̂̂$\hat{Y}=0.115504X+$0.0032384 | | | | | |  |
|  |  |  |  |  | R^2^= 0.9985 | | | |  |  |
|  | | | |  |  |  |  |  |  |  |
| **X** | ${(Y-\hat{Y})}^{2}$ | | | | ${(Y-\bar{Y})}^{2}$ | | | ${(\bar{Y}-\hat{Y})}^{2}$ | | |
| 0.2 | 1.40E-05 | 4.47E-06 | 5.12E-05 | | 3.12E-05 | 7.47E-08 | 2.82E-05 | 3.39E-06 | 3.39E-06 | 3.39E-06 |
| 1.0 | 1.66E-04 | 3.87E-05 | 7.52E-04 | | 3.92E-04 | 4.90E-07 | 4.20E-04 | 4.79E-05 | 4.79E-05 | 4.79E-05 |
| 5.0 | 2.66E-05 | 1.02E-03 | 3.38E-03 | | 7.08E-04 | 4.00E-08 | 6.97E-04 | 1.01E-03 | 1.01E-03 | 1.01E-03 |
| 10.0 | 1.86E-02 | 1.20E-04 | 2.03E-02 | | 2.02E-02 | 2.81E-05 | 1.87E-02 | 3.21E-05 | 3.21E-05 | 3.21E-05 |
| 20.0 | 6.84E-02 | 1.68E-02 | 4.67E-06 | | 1.74E-02 | 1.00E-08 | 1.74E-02 | 1.68E-02 | 1.68E-02 | 1.68E-02 |
| Residual error sum  squares (Eq. 1)  Pure error sum squares (Eq. 2)  Lack-of-fit error sum  squares (Eq. 3) | $\mathrm{SS}_{r}$= $\sum_{i=1}^{I} \sum_{j=1}^{j_{i}} {(Y_{\mathrm{ij}}-\hat{Y_{i}})}^{2}$  (Eq. 1) | | | | $\mathrm{SS}_{\varepsilon}$= $\sum_{i=1}^{I} \sum_{j=1}^{j_{i}} {(Y_{\mathrm{ij}}-\bar{Y_{i}})}^{2}$  (Eq. 2) | | | $\mathrm{SS}_{\mathrm{lof}}$= $\sum_{i=1}^{I} \sum_{j=1}^{j_{i}} {(\bar{Y_{i}}-\hat{Y_{i}})}^{2}$  (Eq. 3) | | |
|  | $\mathrm{SS}_{r}$=1.30e-01 | | | | $\mathrm{SS}_{\varepsilon}$=7.60e-02 | | | $\mathrm{SS}_{\mathrm{lof}}$=5.37e-02 | | |
| Degrees of freedom (DF) | $\mathrm{DF}_{r}$=IJ-2=15-2=13 | | | | $\mathrm{DF}_{\varepsilon}$=IJ-I=15-5=10 | | | $\mathrm{DF}_{\mathrm{lof}}$=I-2=5-2=3 | | |
| Associated variances  (${}^{2}=SS/DF$) | ${}_{r}^{2}=$9.98e-03 | | | | ${}_{\varepsilon}^{2}=$7.60e-03 | | | ${}_{\mathrm{lof}}^{2}=$1.79e-02 | | |
| Fisher ratio (F=${}_{\mathrm{lof}}^{2}$/${}_{\varepsilon}^{2}$)  calculated (if  F_calculated_ < F_tabulated_  then Linear) | 2.36(calculated) <3.71 (tabulated at the 95% with 3 and 10 degrees of freedom) | | | | | | | | | |
| Conclusions | R^2^= 0.9985, while F_calculated_ < F_tabulated_, indicating that the curve is linear | | | | | | | | | |
