# Supplementary material for: A Fragmentation behavior-guided UHPLC-Q-Orbitrap HRMS method for the quantitative analysis of 26 perfluoroalkyl substances and their alternatives in water
Source: PLoS One. 2025 Nov 3;20(11):e0335264. doi: 10.1371/journal.pone.0335264 (PMC12582490; doi:10.1371/journal.pone.0335264)

**Fig S1.** Representative chromatographic separation of 10 target PFAS compounds under optimized conditions. Chromatographic peaks and retention times are shown for each PFAS monitored in their specific ion channels.
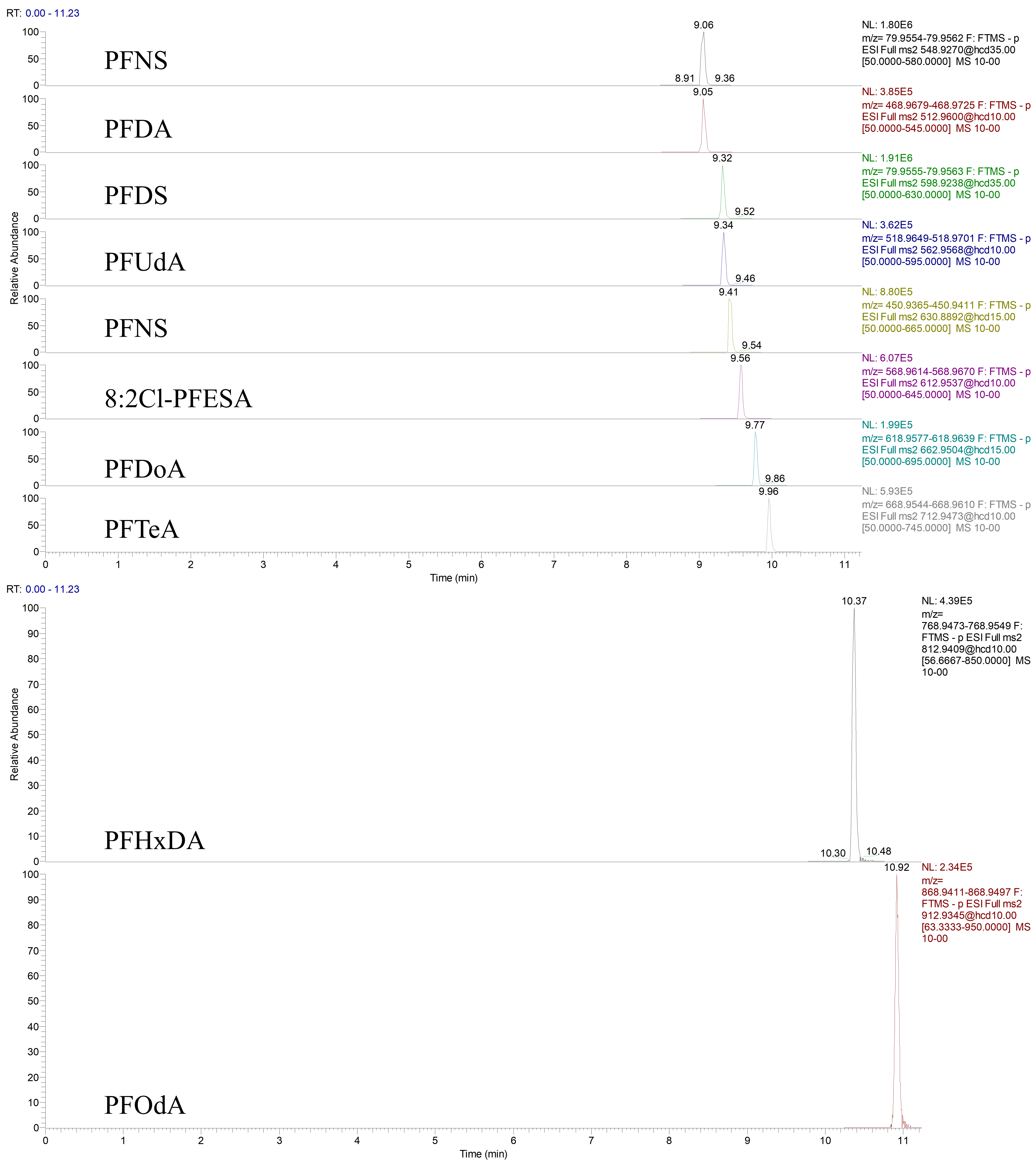

Supplement: S1 Fig — Chromatographic peaks and retention times are shown for each PFAS monitored in their specific ion channels. (DOCX) [file pone.0335264.s004.docx]
