# Supplementary figures and images for: A Fragmentation behavior-guided UHPLC-Q-Orbitrap HRMS method for the quantitative analysis of 26 perfluoroalkyl substances and their alternatives in water
Source: PLoS One. 2025 Nov 3;20(11):e0335264. doi: 10.1371/journal.pone.0335264 (PMC12582490; doi:10.1371/journal.pone.0335264)

**Fig S2**. MS/MS fragmentation pattern and proposed cleavage pathway of PFDS.
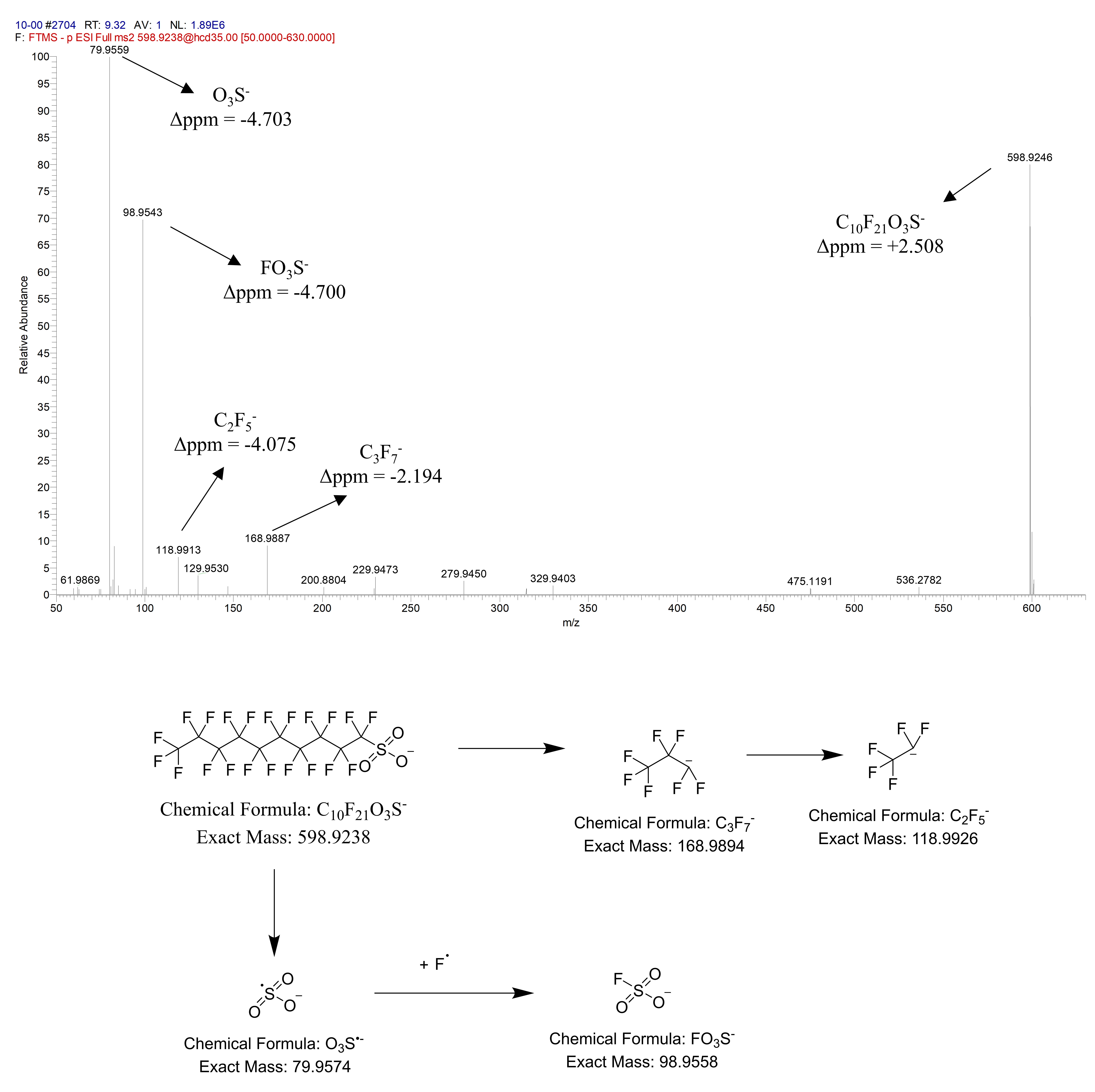

Supplement: S2 Fig — (DOCX) [file pone.0335264.s005.docx]
