# Supplementary material for: A Fragmentation behavior-guided UHPLC-Q-Orbitrap HRMS method for the quantitative analysis of 26 perfluoroalkyl substances and their alternatives in water
Source: PLoS One. 2025 Nov 3;20(11):e0335264. doi: 10.1371/journal.pone.0335264 (PMC12582490; doi:10.1371/journal.pone.0335264)
Supplement: S3 Fig — (DOCX) [file pone.0335264.s006.docx]

**Figure S3**. Illustrative example using PFOS in Orbitrap HRMS: Comparison of mass spectra acquired in (a) SIM mode, (b) PRM mode, and (c) Full-MS mode.
